# Antibody-drug conjugates in breast cancer: redefining targeted therapy

**DOI:** 10.1172/JCI207630

**Published:** 2026-10-01

**Authors:** Chenxu Guo, Leif W. Ellisen

**Affiliations:** 1 Mass General Brigham Cancer Institute, Boston, Massachusetts, USA.; 2 Harvard Medical School, Boston, Massachusetts, USA.; 3 Ludwig Center at Harvard, Boston, Massachusetts, USA.

## Abstract

Antibody-drug conjugates (ADCs) have transformed the treatment landscape of breast cancer and redefined the conceptual distinction between targeted therapy and conventional chemotherapy. Originally conceived as “magic bullets” that selectively deliver cytotoxic warheads to antigen-expressing tumor cells, clinical and mechanistic evidence indicates that ADC activity depends on a broader interplay of target-dependent and target-independent mechanisms, including extracellular payload release, bystander killing, off-tumor uptake, and immune modulation. Here, we examine ADCs in breast cancer as a distinct therapeutic paradigm. We discuss how antigen biology, linker chemistry, payload features, and drug-to-antibody ratio collectively determine efficacy, toxicity, and therapeutic index. We then compare currently approved and emerging HER2- and TROP2-directed ADCs, highlighting how differences in linker stability, payload pharmacology, and bystander capacity can affect clinical outcomes in ADCs sharing the same target. We further discuss the biological basis and translational challenges of de novo and acquired resistance related to targets, payloads, and tumor microenvironmental constraints, as well as the implications of these mechanisms for biomarker development, sequencing rationales, and combination strategies with immune checkpoint inhibitors and DNA repair–targeting therapies. Finally, we outline future directions of ADC development, including expansion of the target space, novel payload modalities, and next-generation antibody and conjugation engineering.

## Introduction

Paul Ehrlich’s century-old “magic bullet” concept envisioned drugs that selectively eradicate diseased cells (1). Antibody-drug conjugates (ADCs) have the potential to realize this vision by coupling highly specific monoclonal antibodies to potent cytotoxic payloads, preferentially directing killing effects to cancer cells. By concentrating potent drugs in tumors while sparing normal tissues, ADCs are designed to expand the narrow therapeutic index of conventional chemotherapies and other bioactive compounds. This effect is achieved through target-selective delivery of a higher maximum tolerated dose (MTD) and use of potent toxins with a lower minimum effective dose that are otherwise too toxic for systemic administration.

Structurally, a typical ADC comprises three core components: a target-specific antibody, a chemical linker, and a cytotoxic small molecule payload (Figure 1). Upon target engagement, ADCs are proposed to operate through a multistep process involving antigen binding, internalization, endolysosomal trafficking, and lysosomal degradation, leading to payload release and ultimately tumor cell death (Figure 1). Despite extensive investigation across preclinical and clinical settings, no consensus exists on optimal design and characteristics of these individual components, and approved breast cancer ADCs span a wide range of successful engineering strategies (2, 3).

The emerging consensus from both preclinical and clinical studies is that ADCs constitute a hybrid modality whose payload delivery is variably target dependent and independent (4). Of note, ADCs are targeted primarily in their biodistribution rather than necessarily in their cytotoxic effects. Following payload release, particularly for membrane-permeable payloads, cytotoxic activity may extend beyond antigen-expressing cells through bystander killing of adjacent cells and extracellular payload diffusion. ADC efficacy and toxicity are governed by unique biological principles including antigen biology, intracellular trafficking, payload pharmacology, and tumor microenvironment (TME) context, motivating a mechanistic and translationally focused assessment to optimize their application for breast cancer therapy.

## Currently approved and emerging ADCs in breast cancer

### Overview of FDA-approved ADCs in breast cancer.

Currently, four ADCs are FDA approved for breast cancer treatment, utilizing two molecular targets, two payload classes, and distinct linkers (Table 1). T-DM1 couples the human epidermal growth factor receptor 2 (HER2) antibody trastuzumab to the tubulin inhibitor emtansine (DM1) via a noncleavable MCC linker, restricting cytotoxicity to HER2-overexpressing cells without bystander activity (5). T-DXd uses a cathepsin-cleavable linker to deliver the topoisomerase 1 inhibitor (TOP1i) deruxtecan (DXd), enabling lysosomal release and membrane-permeable diffusion to neighboring cells (6). SG targets tumor-associated calcium signal transducer 2–expressing (TROP2-expressing) cells with a pH-sensitive hydrolyzable linker to deliver the TOP1i SN-38, generating sustained intracellular and extracellular payload release within the TME (7, 8). Dato-DXd also targets TROP2 but employs a cleavable linker with DXd, conferring bystander capacity analogous to T-DXd (9, 10).

Notably, T-DXd, Dato-DXd, and SG share TOP1i payloads with cleavable or hydrolyzable linkers, a commonality in design that confers bystander killing through extracellular release of membrane-permeable payloads. These ADCs span a broad range of drug-to-antibody ratios (DARs) (~4 to ~8), suggesting that optimized linker-payload chemistry can preserve tolerability and therapeutic index despite varying DAR.

### HER2-directed ADCs: a shared target and divergent designs.

T-DM1 and T-DXd exemplify how ADCs sharing the same antibody diverge in efficacy and toxicity due to differences in linker cleavability, payload type, and bystander capacity. While T-DM1 became a standard treatment for HER2-positive disease through the EMILIA study in the metastatic setting (11) and the KATHERINE trial in the post-neoadjuvant setting (12), T-DXd was subsequently proved to be generally more effective. In head-to-head trials, T-DXd dramatically outperformed T-DM1 for both HER2-positive metastatic disease (DESTINY-Breast03; median progression-free survival [PFS] 25.1 vs. 7.2 months) (13) and post-neoadjuvant residual disease (DESTINY-Breast05; 3-year invasive disease–free survival 92.4% vs. 83.7%) (14). The mechanistic basis for T-DXd’s superiority to T-DM1 is likely multifactorial: its higher DAR delivers more payload per antibody molecule; its DXd payload is more potent than DM1’s; and its cleavable linker with membrane-permeable payload confers bystander killing that T-DM1 lacks (6, 15).

The success of T-DXd has also expanded the scope of HER2-directed therapy beyond clinically HER2-positive disease to tumors with lower levels of HER2 expression that lack HER2 oncogene addiction. Indeed, T-DXd demonstrated significant PFS and overall survival (OS) benefits compared with chemotherapy in HER2-low (DESTINY-Breast04) (16) and HER2-ultralow (DESTINY-Breast06) (17) breast cancers. Meanwhile, newer HER2-ADCs currently in trials illustrate persistent trade-offs between efficacy and distinct toxicities of these agents. Trastuzumab duocarmazine improved PFS over physician’s choice therapy in pretreated HER2-positive disease in the TULIP trial but faces ocular toxicity challenges (18). Another HER2-ADC, ARX788, showed clinical viability with improved PFS over lapatinib-capecitabine in the ACE-Breast-02 trial (19). Finally, the third-generation TOP1i-based trastuzumab pamirtecan is reported to have met its phase III primary PFS endpoint against T-DM1, though its peer-reviewed efficacy and safety details remain to be reported (20).

### TROP2-directed ADCs: extending targeted therapy.

Treatment of triple-negative breast cancer (TNBC), a breast cancer subtype historically lacking in target-selective therapies, has been revolutionized by the development of ADCs (21). SG was the first ADC displaying a significant OS benefit in pretreated metastatic TNBC (mTNBC). In the pivotal ASCENT trial, SG improved median PFS (5.6 vs. 1.7 months) and OS (12.1 vs. 6.7 months) compared with chemotherapy (22). The SG indication has since expanded to earlier treatment lines based on the subsequent ASCENT-03 study (23), while ASCENT-04 demonstrated PFS benefits when SG plus anti–programmed cell death protein 1 (anti–PD-1) pembrolizumab was compared with chemo-pembrolizumab in first-line anti–programmed cell death ligand 1–positive (anti–PD-L1–positive) TNBC (24). In addition, SG showed significant PFS (25) and OS (26) benefits over chemotherapy in advanced hormone receptor–positive (HR-positive)/HER2-negative breast cancer.

Similar clinical progress has been achieved with the other approved TROP2-directed ADC, Dato-DXd. In TROPION-Breast01, Dato-DXd improved PFS over chemotherapy in pretreated HR-positive/HER2-negative disease (27). Final OS analysis did not reach statistical significance, potentially confounded by postprogression crossover to other ADCs (28). Dato-DXd is also approved for first line treatment of metastatic TNBC based on results of the phase 3 TROPION-Breast02 trial (29). As with HER2-directed ADCs, emerging TROP2-ADCs show potential promise. Sacituzumab tirumotecan employs an innovative dual-cleavage linker and belotecan-derived TOP1i payload and achieved notable PFS (6.7 vs. 2.5 months) and objective response rate (ORR; 45.4% vs. 12.0%) improvements over chemotherapy in pretreated mTNBC in the phase III OptiTROP-Breast01 trial (30).

### Toxicity patterns and implications.

Individual ADCs typically demonstrate distinct toxicity signatures reflecting a composite of their structural and pharmacological properties rather than that of the payload alone. A recent meta-analysis correlates linker cleavability, DAR, and free payload levels with hematologic toxicity severity (31). Such hematologic toxicity reflects systemic payload exposure from premature linker cleavage in circulation, nonspecific uptake by hematopoietic progenitors, and target-independent FcγR-mediated internalization by myeloid cells (32, 33). SG predominantly causes neutropenia and diarrhea, consistent with its distinct linker chemistry and payload mechanism (22, 34). By contrast, T-DXd, with its more stable peptide cleavable linker, exhibits roughly half the rate of neutropenia seen with SG; instead, its most insidious toxicity is interstitial lung disease (ILD), a potentially life-threatening complication occurring in up to 15% of patients (35). Although generally poorly understood, ILD is a target-independent effect rather than an on-target consequence, driven by ADC uptake in alveolar macrophages rather than HER2-expressing lung epithelium (36). T-DM1 exhibits its unique profile of thrombocytopenia and hepatotoxicity, driven by megakaryocyte uptake via FcγRIIa binding or micropinocytosis (37, 38) and hepatic ADC processing (39, 40), respectively. Since the precise mechanisms driving these varied target-independent toxicities are not fully understood, rational ADC design to “engineer out” dose-limiting toxicities remains an ongoing challenge.

Beyond mechanistic understanding, proactive toxicity monitoring and management are increasingly important for maximizing the therapeutic index of ADCs in clinical practice. For TOP1i-based ADCs such as T-DXd and SG, nausea and vomiting often require guideline-directed antiemetic prophylaxis, while early recognition of ILD/pneumonitis and prompt corticosteroid treatment are critical for preventing severe outcomes (41). Ocular toxicities associated with certain ADCs may benefit from ophthalmologic monitoring and timely supportive interventions (18). Together, these observations underscore that many ADC-associated toxicities are manageable when recognized early and addressed through multidisciplinary supportive care (41).

## Structure, design principles, and mechanisms of action

### Target antigen biology.

Effective ADC target selection extends beyond surface expression level. Traditional, ideal antigens combine high tumor specificity with efficient internalization kinetics, including rapid endocytic uptake, lysosomal trafficking, and limited shedding of extracellular domains that could sequester circulating ADCs (42, 43). Specifically, antigen recycling further increases payload delivery, as repeated cycles of internalization and resurfacing enable a single antigen molecule to shuttle multiple ADC molecules into the cell (44). HER2 and TROP2 exemplify such targets, combining high expression with rapid internalization and recycling to support intracellular payload accumulation (42, 43, 45).

However, not all ADC design strategies depend on target antigen internalization. A distinct class of noninternalizing ADCs in clinical development targets extracellular antigens or stromal components, such as tumor-associated extracellular matrix proteins and secreted factors such as Gal-3BP (46). These targets are stably displayed on the tumor cell surface or within the peritumoral space but do not undergo efficient endocytic uptake (47). Rather than relying on lysosomal processing, noninternalizing ADCs exploit extracellular linker cleavage or local pH changes within the TME to release membrane-permeable payloads that diffuse into surrounding tumor cells (48–50). This strategy expands ADC targets beyond antigens with favorable internalization kinetics and may be particularly relevant in breast cancer, where computational analyses have identified such candidate targets that exhibit suitable tumor-selective expression (51).

Intra- and intertumoral antigen heterogeneity restrains conventional ADC efficacy. However, this constraint can be partially mitigated via bystander effects of ADCs with cleavable linkers and membrane-permeable payloads. For instance, due to its limited bystander effect, T-DM1 is highly effective in HER2-positive breast cancer but lacks meaningful clinical activity in HER2-low or HER2-ultralow populations where heterogeneity may play a larger role (52, 53). By contrast, T-DXd can successfully eliminate adjacent HER2-negative cells in HER2-heterogeneous tumors (15, 49). Clinically, the DESTINY-Breast04 trial established substantial T-DXd survival benefits in HER2-low patients previously excluded from HER2-targeted therapies (16). However, bystander effects may also decrease tumor cell spatial specificity and enhance off-target toxicity, highlighting ADCs’ dual identity as targeted yet systemic therapies.

### Payloads as mechanistic drivers.

As noted, approved ADCs for breast cancer employ two dominant payload classes: tubulin-binding agents (e.g., DM1) and DNA-targeting TOP1i agents (e.g., DXd and SN-38) (4). One element of the success of TOP1i-associated ADCs in breast cancer is the fact that these drugs are not typically used as standard systemic chemotherapy. Since tumors are treatment naive to this payload class, cross-resistance related to prior therapy is limited (54).

While ADC payloads share cytotoxic mechanisms with conventional chemotherapy, their intracellular exposure kinetics differ due to antibody-mediated delivery and lysosomal processing compared with systemically administered free drug (2). ADC antitumor efficacy heavily depends on payload potency and mechanisms of action: the more favorable profile of DXd over DM1 likely contributes to the clinical superiority of T-DXd over T-DM1 despite incorporation of the same antibody component (6). Beyond potency, payload class shapes the broader tumor response, as exemplified by TOP1i agents that engage the DNA damage response and induce immunogenic cell death (ICD), creating potential synergies with immune checkpoint inhibitors (ICIs) and DNA repair–targeting therapies (4). Critically, payload membrane permeability dictates bystander killing capacity. Permeable payloads like DXd and SN-38 can diffuse across cell membranes to eliminate antigen-negative neighbors, whereas charged, impermeable DM1 is confined to the originally targeted cell. Increased permeability may overcome heterogenous target expression in breast cancer, further explaining why T-DXd outperforms T-DM1 despite targeting the same antigen (15, 55).

### Linkers and DAR.

Linker chemistry governs the site and timing of payload release, fundamentally shaping both efficacy and off-tumor toxicity. Noncleavable linkers as in T-DM1 require complete antibody degradation in the lysosome, restricting payload cytotoxicity to antigen-positive cells. Cleavable linkers, by contrast, liberate payloads via protease cleavage (T-DXd and Dato-DXd), acidic pH (SG), or reducing conditions, allowing subsequent diffusion and bystander killing (56). Additionally, pH-sensitive hydrolyzable linkers enable gradual extracellular payload release within the acidic TME even in the absence of internalization, providing sustained local drug exposure (8). Recent evidence further indicates that TME extracellular proteases can cleave cathepsin-sensitive peptide linkers prior to internalization, potentially contributing to ADC activity even without direct antigen engagement (57).

The DAR critically influences ADC structural homogeneity, aggregation propensity, and efficacy. While high DAR increases payload delivery, it elevates overall ADC hydrophobicity, promoting aggregation, accelerating clearance, and reducing tolerability (58). The “saturation front” hypothesis further highlights DAR trade-offs: high-DAR ADCs rapidly saturate perivascular tumor regions, creating steep concentration gradients that impede deep tumor penetration and thus reduce efficacy despite higher payload delivery per antibody (59). In preclinical studies, coadministering unconjugated antibodies can improve penetration by competing for surface binding, effectively reducing the local DAR and overcoming the saturation front problem (60). Clinical analyses align with these findings, as lower DAR ADCs permit dose escalation and correlate with improved response rates, supporting a therapeutic index advantage (61).

## Current challenges and pressing translational questions

The exact mechanisms and optimal design characteristics of ADCs remain actively debated (2). On one hand, preclinical and clinical evidence suggests that target-dependent delivery of ADC is at least partially required for efficacy. On the other hand, on-target, on-tumor delivery is not the sole driver of ADC activity. Pharmacodynamic analyses suggest ADCs do not increase the MTD of conventional chemotherapy, while pharmacokinetic analyses suggest on-tumor ADC distribution is typically less than 1% of the administered dose (62). Beyond bystander effects, uptake of ADCs by either receptor-dependent (FcγRs, FcRn, and C-type lectin receptors) or receptor-independent (nonspecific endocytosis) mechanisms also contributes to off-target toxicity in normal cells (32, 37). Meanwhile, ADCs also actively modulate the immune landscape through engagement of FcγRs to drive antibody-dependent cellular phagocytosis and payload-induced ICD and myeloid cell activation through TLR4 and STING pathways (57). The sections below discuss ADC features driving response and resistance, mechanisms of acquired resistance, and rational therapeutic combination strategies to overcome them (Figures 2 and 3).

### Determinants of ADC response and de novo resistance.

Target expression variably predicts response to HER2-directed ADCs (53) (Figure 2). For T-DM1, higher HER2 abundance correlates with significantly prolonged PFS and improved ORR (52). In the DAISY trial, T-DXd response duration correlated with HER2 expression (63), and resistance emerged through HER2 loss and *ERBB2* binding site mutations that impair ADC internalization and increase IC_50_ (64). Analogous findings have been reported for SG: TROP2 expression correlates to some degree with benefit in mTNBC (65), while an acquired *TROP2* mutation that disrupts antibody binding has been associated with resistance (66). For Dato-DXd, clinical predictive utility remains to be established, though preclinical models suggest that higher TROP2 expression correlates with response and restores sensitivity in resistant tumors (67).

An additional challenge is that target antigen expression is not static but evolves during therapy. Exposure to HER2-directed therapies, endocrine therapy, and chemotherapy may alter antigen density through selective pressure, transcriptional reprogramming, and clonal evolution, potentially shifting tumors between HER2-zero, HER2-low, and HER2-positive states (64, 68, 69). Such temporal variability complicates patient selection for ADC treatment when therapeutic decisions rely on archival tissue samples, as antigen assessment needs to be viewed as a dynamic rather than fixed tumor characteristic.

Beyond antigen expression, ADC efficacy hinges on intracellular payload trafficking, processing, and cellular vulnerability. De novo resistance can emerge from defective lysosomal processing, even when target expression is preserved. For instance, preclinical models show T-DM1–resistant HER2-positive cells have normal internalization but elevated lysosomal pH, which impairs proteolytic payload release (70). Additionally, loss of the SLC46A3 transporter prevents DM1 transfer to cytoplasm (71, 72), while upregulated caveolin-1 reroutes ADCs away from productive lysosomal pathways via caveolae-mediated endocytosis (73, 74). Moreover, the disproportionate success of ADCs in breast cancer compared with several other solid tumor types is not strictly related to differential target expression but more likely to the disease’s high intrinsic chemosensitivity (75).

Furthermore, heterogenous tumor-intrinsic mechanisms governing cell cycle progression, DNA repair, and apoptosis fundamentally dictate payload sensitivity within breast cancer (76). For example, aberrant STAT3 activation confers T-DM1 resistance in HER2-positive breast cancer (77). Multiomic profiling identified the mitotic kinase PLK1 as a primary driver of both de novo and acquired resistance to T-DM1, correlating with accelerated cell cycle progression and enhanced DNA repair (78). Inhibiting PLK1 overcomes T-DM1 resistance in preclinical models by triggering mitotic arrest, caspase activation, and DNA damage through CDK1-dependent phosphorylation and inactivation of BCL-2/BCL-xL (78). Finally, drug efflux transporters are known to mediate payload resistance. In a large cohort of T-DXd–treated patients, lower expression of ABCC1, which effluxes DXd, correlated with longer OS independently of HER2 status (79), while increased ABCC1 has been identified as a primary mediator for T-DM1 resistance in preclinical models (80).

Though less clinically validated than tumor-intrinsic determinants, the TME is increasingly recognized as a modulator of ADC response and resistance through pharmacokinetic and immunological mechanisms (Figure 3). The breast cancer TME is generally permissive to macromolecular delivery, lacking the extreme desmoplasia, poor or disordered vascularization, and profound immunosuppression that restrict ADC penetration in stroma-dense tumors such as pancreatic ductal adenocarcinoma (81, 82). Furthermore, extracellular proteases such as cathepsin L secreted by both tumor and stromal cells in the breast cancer TME can actively drive ADC efficacy, as discussed above (57). In contrast, physical barriers within the TME can impose restrictions on ADC distribution. As ~150 kDa macromolecules, ADCs are hindered by dense extracellular matrices, high interstitial fluid pressure, and the binding site barrier. In xenografts, transient competitive inhibition improved T-DM1 penetration into deeper tumor tissues and enhanced efficacy, as high-affinity antigen binding near perivascular regions creates the “saturation front” problem that limits ADC infiltration (83).

Finally, the TME immune context adds further complexity. While direct clinical evidence in breast cancer remains limited, syngeneic TNBC models reveal that TROP2 actively shapes physical barriers to T cell infiltration, driving immune exclusion via claudin-7–associated tight junction regulation (84). Preclinically, T-DXd induces ICD, promoting DC activation and durable immune memory responses (85). However, biomarker analyses from the DAISY trial found no significant increase in granzyme B–positive CD8^+^ T lymphocytes in patient biopsies during T-DXd treatment, indicating that ICD-mediated immune activation may be less robust in patients than preclinical models predict (86). These findings highlight an important translational gap between preclinical demonstrations of ADC-induced immunogenicity and clinically measurable immune activation, warranting caution when extrapolating preclinical ICD signals to the clinical efficacy of ADC–ICI combinations.

These various ADC response determinants have not yet been successfully developed as clinical biomarkers, and current target-based patient selection in breast cancer has notable limitations. For T-DXd, HER2 immunohistochemistry (IHC) suffers from interobserver variability, spatiotemporal heterogeneity, and a categorical scoring system originally designed for trastuzumab eligibility rather than for ADCs that shows activity across a broader expression continuum (87). Similarly, TROP2 IHC fails to reliably predict clinical benefit. In the TROPiCS-02 trial, SG improved OS regardless of TROP2 expression, suggesting static target expression–based selection alone is insufficient (26). Emerging approaches to target assessment include quantitative immunofluorescence, spatial antigen mapping, and AI-assisted digital pathology for standardized, observer-independent scoring (88).

### Acquired resistance mechanisms and implications for ADC sequencing.

Antigen downregulation is a recurrent mechanism associated with acquired ADC resistance in the clinic. Among patients treated with HER2-directed ADCs, 49% showed HER2 downregulation in paired patient samples following T-DXd progression, with over half of these experiencing complete HER2 loss, which correlates with reduced ADC internalization and increased IC_50_ in isogenic models (64). Alternatively, resistance can emerge via acquired HER2 binding site mutations (V597M and P593R) despite preserved surface expression. These mutations reduce trastuzumab binding, impairing ADC internalization through pharmacological rather than transcriptional mechanisms (64). Importantly, resistance driven by either of these mechanisms can be overcome using a TROP2-directed ADC carrying the same DXd payload, providing a strong rationale for antigen-switching sequencing strategies in these cases (64). Target-mediated acquired resistance also affects TROP2-directed ADCs. In a TNBC patient with a prolonged response and subsequent acquired resistance to SG, postprogression analysis identified a novel TROP2 T256R missense mutation in the dominant resistant clone (66). This single point mutation conferred SG resistance by disrupting TROP2’s plasma membrane localization and reducing cell surface binding to the hRS7 antibody (66).

Acquired payload target mutations represent another clinical mechanism driving ADC resistance. In breast cancer patients progressing on TOP1i-based ADCs, circulating tumor DNA analysis in a small series detected distinct TOP1 missense mutations in 12.9% of cases versus 0.7% in ADC-naive patients treated for breast cancer (89). These mutant proteins exhibited reduced enzymatic activity, attenuated covalent DNA binding, and functional resistance to both DXd and SN-38 payloads, and patients harboring TOP1 mutations experienced dramatically shorter responses to subsequent TOP1i-based ADCs (median 52 vs. 455 days) (89). Another acquired resistance pathway involves upregulation of the ABCC1 drug efflux pump via the NFE2L2/KEAP1 axis, whereby KEAP1 negatively regulates the NFE2L2 transcription factor that drives ABCC1 expression. Post–T-DXd tumor specimens showed enriched mutations in NFE2L2 and KEAP1 relative to T-DXd–naive samples, suggesting transcriptional upregulation of DXd efflux is actively selected for under ADC pressure, driving resistance independently of target antigen loss (79).

For patients who typically receive multiple lines of therapy in the metastatic setting, the question of optimal ADC timing relative to chemotherapy has been addressed by comparisons across trials. Results from DESTINY-Breast04 (second-line T-DXd in HER2-low) and DESTINY-Breast06 (first/second-line T-DXd in HER2-low/ultralow) collectively suggest that earlier ADC deployment produces longer PFS, potentially suggesting that preserved target expression and reduced preexisting payload resistance at earlier treatment lines favor ADC efficacy (16, 17). By contrast, defining the optimal sequencing strategy for use of multiple ADCs remains a central, unresolved clinical challenge. While the goal is to rationally switch ADC targets or payloads to preserve efficacy, current evidence is largely retrospective and highly heterogeneous, revealing substantial but variable cross-resistance (90). Despite these limitations, several provisional sequencing principles have emerged, including earlier deployment of effective ADCs and switching payload classes or antigen targets when feasible (Table 2). A multicenter study found that switching to a second ADC (ADC2) with a different payload class yields higher response rates (~22.6% ORR) than same-payload sequences (~5.3% ORR), but PFS for ADC2 remained similar regardless of payload class (91). Similarly, real-world analyses indicate that results with sequential use of currently approved ADCs are generally unsatisfactory, as time to treatment failure and PFS are consistently shorter for ADC2 regardless of sequence order, HR status, or intervening chemotherapy (92–94).

Nonetheless, cross-resistance appears most pronounced when sequential ADCs share payload mechanisms. A database analysis of post–T-DXd patients showed that subsequent SG yielded inferior real-world PFS across subtypes, supporting the rationale to switch to non-TOP1i therapies after T-DXd progression rather than substituting another TOP1i-based ADC (95). Conversely, switching payloads while retaining the same target has proven effective for use of T-DXd after T-DM1 exposure. In the DESTINY-Breast02 trial, T-DXd produced a median PFS of 17.8 versus 6.9 months and an ORR of 70% versus 29% over chemotherapy after prior T-DM1 treatment (96), consistent with the absence of cross-resistance between maytansinoid and TOP1i payloads. Furthermore, despite frequent ADC2 resistance, a clinically meaningful subset still achieves durable responses, including patients who switch antibody targets (97), experience prolonged initial ADC responses, or receive ADC2 in an earlier treatment line (98). Ongoing and impending clinical trials have been designed to systematically test sequencing and biomarkers for currently approved ADCs. Nonetheless, innovation and implementation of new ADC classes with distinct targets and payloads are needed to increase options and improve outcomes associated with sequential ADC use.

### Combination strategies to enhance response and overcome resistance.

The rationale for combining ADCs with ICIs centers in part on the ability of TOP1i payloads to induce ICD, thereby priming an immune response that can be further augmented via ICI (99). Payloads including DXd and SN-38 trigger ICD hallmarks such as calreticulin surface exposure, high mobility group protein B1 release, and ATP secretion that promote antigen cross-presentation and T cell priming, while ICIs reinvigorate exhausted T cells to amplify the adaptive immune response (100–102). Potential synergy is supported by compelling preclinical data, as the combination of T-DM1 with dual immune checkpoint blockade nearly eradicates tumors in HER2-positive models (103). Clinically, TROP2-directed SG plus the anti–PD-1 antibody pembrolizumab demonstrated superior PFS compared with chemotherapy plus pembrolizumab for first-line treatment of PD-L1–positive advanced TNBC in the randomized phase III ASCENT-04 trial (24). Similarly, in the phase Ib/II BEGONIA trial, first-line Dato-DXd plus durvalumab achieved approximately 80% ORR regardless of PD-L1 expression (104). Clinical data supporting the addition of an ICI to HER2-directed ADCs have to date been less convincing. In the phase Ib DS8201-A-U105 study, T-DXd plus nivolumab achieved a 66% ORR in pretreated HER2-positive metastatic breast cancer (105). This is comparable to historical T-DXd monotherapy data, which showed ORRs of 61% in DESTINY-Breast01 (106), 70% in DESTINY-Breast02 (96), and 80% in DESTINY-Breast03 (13). Similarly, T-DXd plus nivolumab achieved 50% ORR in HER2-low breast cancer, which is again comparable to T-DXd monotherapy in DESTINY-Breast04 (52%) (16). As with other immunotherapy applications in oncology, improved predictive markers and alternative immune activation strategies remain unmet needs.

The hypothesized synergy between TOP1i-based ADCs and PARP inhibitors (PARPis) operates through three convergent mechanisms: (a) PARP1 normally PARylates TOP1 cleavage complex (TOP1cc) to release it from DNA, while PARP inhibition prevents this release, stabilizing TOP1cc and prolonging DNA damage; (b) PARP inhibition blocks tyrosyl-DNA-phosphodiesterase 1, the phosphodiesterase required to excise covalently trapped TOP1 from DNA, further preventing TOP1cc repair; and (c) PARP inhibition impairs repair of replication-associated double-strand breaks that arise when replication forks collide with TOP1i-stabilized TOP1cc (107).

This combination was initially tested in 6 patients (SEASTAR trial), where SG plus the PARPi rucaparib demonstrated antitumor activity regardless of homologous recombination mutation status or PARPi preexposure (108). A subsequent clinical trial evaluated the hypothesis that ADC-mediated delivery of the TOP1i creates a high tumor/normal cell dose gradient that enables a temporal window for sequential rather than concurrent ADC/PARPi dosing. In the phase Ib trial of SG plus PARPi talazoparib in mTNBC, this strategy was shown to mitigate the dose-limiting myelosuppression that has plagued systemic TOP1i/PARPi combinations (109). Further testing of this strategy is likely to involve newer PARP1-selective inhibitors, as currently approved PARPis inhibit both PARP1 and PARP2, while it is PARP2 that disproportionately drives hematological toxicity (110).

## Future generations of ADCs in breast cancer

### Expanding the target space.

ADC target development in breast cancer is expanding beyond HER2 and TROP2 toward both tumor-associated antigens and nontumor components of the TME (Figure 4). To overcome the limitations of heterogeneous antigen expression and acquired resistance, a diverse array of novel tumor-associated membrane proteins is under active preclinical and clinical investigation (Table 3). While these novel targets offer distinct tumor selectivity and internalization kinetics, they also warrant rigorous evaluation of their respective safety and efficacy profiles. An alternative, emerging paradigm shifts ADC targeting from tumor cells to the TME (111). Utilizing noninternalizing ADCs with cleavable linkers directed against nontumor cells circumvents the need for uniform tumor antigen expression and relies on extracellular payload release within the TME (47). Key targets include cancer-associated fibroblasts and endothelia, allowing for localized payload release within the TME to effectively ablate tumor-promoting stromal and vascular compartments as well as adjacent breast cancer cells (Table 3).

### New payload modalities.

Payload modality innovation represents a major frontier in ADC design (Figure 4 and Table 4). Degrader-antibody conjugates merge targeted delivery with proteolysis-targeting chimeras or molecular glues to eliminate oncoproteins via the ubiquitin-proteasome system and have shown sensitive and specific efficacy in breast cancer models (112, 113). Another approach utilizes immune-stimulating antibody conjugates (ISACs) to deliver immune agonists and convert immunologically cold breast tumors into inflamed niches, promoting localized myeloid activation and T cell priming (114). However, clinical translation remains challenging; despite strong mechanistic rationales, ISACs inherently suffer from a narrow therapeutic index, as maximizing antitumor potency frequently exacerbates immune-related systemic toxicity (114, 115).

In addition, several other novel payload modalities remain primarily in proof-of-concept stages (115). These include transcription-directed payloads that exploit cytotoxicity outside the canonical tubulin or TOP1 axes to kill nondividing tumor cells through transcriptional arrest. Antibody-oligonucleotide conjugates extend the ADC concept to enable sequence-specific knockdown of oncogenic transcripts or resistance-mediating genes (116). Immunotoxins, an exploratory class fusing targeting antibodies to bacterial toxins, eradicate both dividing and nondividing tumor cells by inhibiting protein synthesis across various breast cancer subtypes (117). Antibody-radionuclide conjugates leverage antibody specificity to deliver localized radiation with theranostic potential, though clinical progress is hindered by dose-limiting myelosuppression, heterogeneous radionuclide distribution, and the absence of prospective randomized trials (118). Finally, antibody-photosensitizer conjugates are activated by focal light to induce targeted ICD and durable antitumor immune memory, providing a strong rationale for combination with immune checkpoint blockade (119).

### Antibody and conjugation engineering.

Antibody and conjugation engineering represents another driver for future ADC design in breast cancer (Figure 4 and Table 5). Biparatopic ADCs, which bind nonoverlapping epitopes of the same antigen, enforce receptor clustering and accelerate internalization, while bispecific ADCs simultaneously engage two independent antigens to mitigate antigen escape and enhance tumor selectivity (120). Additionally, dual-payload ADCs address tumor heterogeneity and acquired resistance by codelivering two distinct cytotoxic warheads with nonoverlapping mechanisms, raising the barrier to resistance and achieving synergistic cytotoxicity (121).

Conjugation technologies are also rapidly evolving. Conventional ADCs rely on stochastic coupling, creating heterogeneous mixtures with variable DARs that complicate pharmacokinetics and clinical consistency (122). By contrast, site-specific conjugation technologies enable precise positional control of payload attachment (123). This approach generates homogeneous ADCs with defined DARs, improving pharmacokinetic consistency, reducing toxicity, enhancing reproducibility, and widening the therapeutic index, as seen in approved ADCs like T-DXd (124).

Moreover, orthogonal engineering strategies enable further spatial and pharmacokinetic control. Masked or “probody” ADCs utilize auto-inhibitory peptides that block antigen binding on normal cells in the TME but are cleaved exclusively by tumor-enriched proteases, thereby sparing normal tissues and widening the therapeutic window (3). Furthermore, Fc backbone engineering provides another dimension for optimization (125), allowing for enhancement of immune-mediated cytotoxicity or extension of plasma half-life for prolonged tumor exposure.

## Concluding perspective

The emerging paradigm of ADCs in breast cancer represents not only an incremental advance in targeted drug delivery but also a conceptual evolution of chemotherapy. Clinical and mechanistic data suggest ADC activity arises from a complex interplay of antibody-mediated distribution, linker stability, payload pharmacology, and microenvironmental release, involving both target-dependent and target-independent mechanisms such as bystander killing and off-tumor uptake. Breast cancer has become the archetype for ADC development, with HER2- and TROP2-directed ADCs transforming treatment paradigms, expanding therapeutic eligibility, and displacing conventional chemotherapy. Realizing next-generation ADC potential will require mechanism-informed biomarkers beyond target-based IHC, biology-driven sequential treatment strategies, rational combinations, and continued innovation in targets, payloads, antibodies, and conjugation. Translational evaluation should focus on improved therapeutic index through better patient selection, adaptive treatment strategies, and reduced systemic and off-tumor toxicity. Together, these priorities define a translational roadmap aligned with the biological complexity of ADC mechanisms.

## Conflict of interest

LWE has received research support from Sanofi.

## Funding support

This work was supported by NIH/NCI grant 1R01CA260890 and NIH/NCI SPORE grant 2P50CA168504 (LWE).

## Figures and Tables

**Figure 1:**
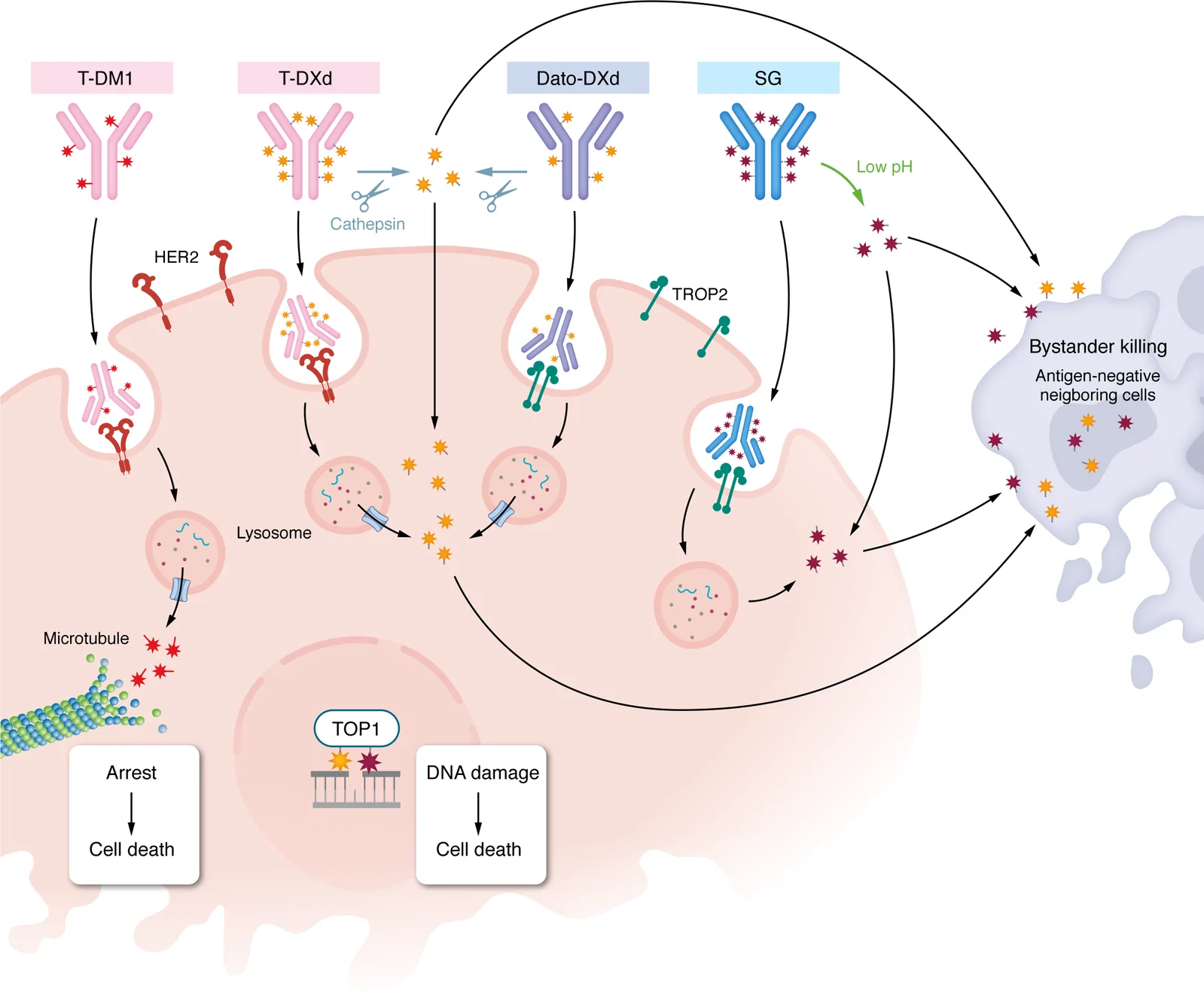
Distinct structural features and mechanisms of action of FDA-approved ADCs in breast cancer. Trastuzumab emtansine (T-DM1), trastuzumab deruxtecan (T-DXd), datopotamab deruxtecan (Dato-DXd), and sacituzumab govitecan (SG) differ in target, linker chemistry, payload class, and drug-to-antibody ratio, which together shape intracellular processing, bystander activity, and downstream cytotoxic effects. T-DM1 carries the noncleavable maytansinoid payload DM1 and primarily requires antigen binding, internalization, lysosomal degradation, and intracellular release of a membrane-impermeable catabolite that disrupts tubulin dynamics and induces mitotic arrest, with minimal bystander killing. In contrast, T-DXd and Dato-DXd contain cleavable tetrapeptide linkers and membrane-permeable topoisomerase 1 inhibitor (TOP1i) payloads that can be released after cathepsin-mediated cleavage, diffuse into neighboring antigen-low or antigen-negative cells, and promote bystander killing. SG similarly delivers a TOP1i payload through a pH-sensitive linker that can undergo hydrolysis in acidic conditions, further supporting extracellular payload liberation and bystander effects.

**Figure 2:**
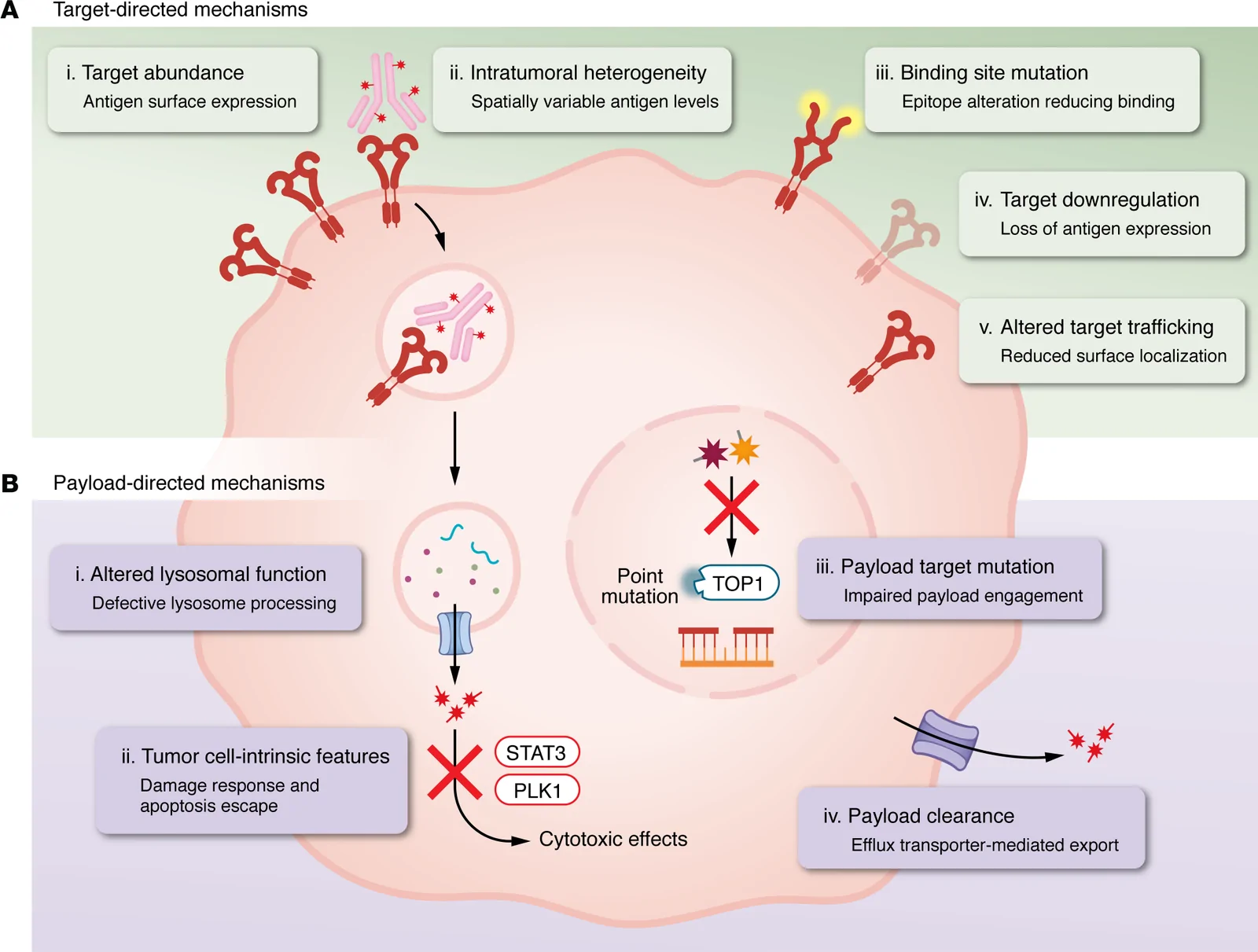
Target- and payload-mediated determinants of ADC response and resistance in breast cancer. ADC activity is governed by coordinated target-directed and payload-related mechanisms that influence response, de novo resistance, and acquired resistance. (**A**) Target-directed mechanisms include antigen surface abundance, intratumoral heterogeneity with spatially variable antigen expression, binding site mutations that alter epitopes and reduce antibody binding, target downregulation resulting in loss of antigen expression, and altered target trafficking that reduces surface localization and limits ADC accessibility. (**B**) Payload-related mechanisms include altered lysosomal function leading to defective ADC processing and payload release, tumor cell–intrinsic features such as growth capacity and apoptosis escape (e.g., via signal transducer and activator of transcription 3 [STAT3] and polo-like kinase 1 [PLK1]), mutations in payload targets (e.g., TOP1) that impair drug engagement, and active payload clearance mediated by efflux transporters. These processes collectively determine intracellular payload delivery and cytotoxic effects, informing biomarker development, treatment sequencing, and rational combination strategies.

**Figure 3:**
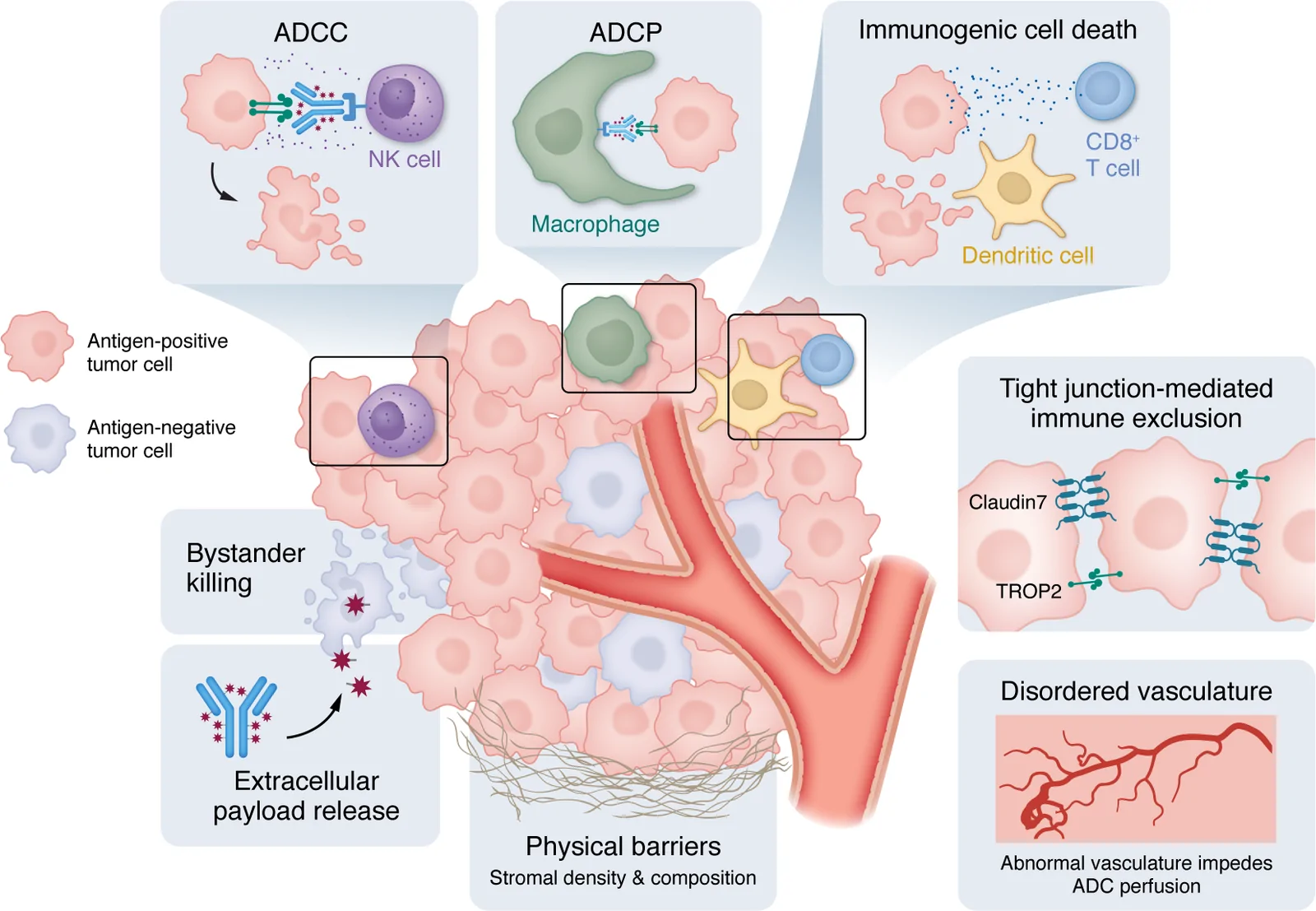
Tumor microenvironmental regulation of ADC activity and resistance. The tumor microenvironment (TME) critically influences ADC distribution, payload activity, and therapeutic outcome. Physical barriers, including stromal architecture and extracellular matrix composition, restrict antibody penetration, while tight junction–mediated immune exclusion (e.g., TROP2 and claudin-7 expression) may further impede lymphocyte infiltration. Disordered tumor vasculature impairs ADC perfusion and distribution compared with normal vasculature. Extracellular payload release, driven by linker instability or extracellular cleavage, can enhance bystander killing of neighboring antigen-negative tumor cells, particularly for membrane-permeable payloads. The immune context contributes through Fc-dependent effector functions, including antibody-dependent cellular cytotoxicity (ADCC) mediated by NK cells and antibody-dependent cellular phagocytosis (ADCP) mediated by macrophages. In addition, ADC-induced immunogenic cell death promotes dendritic cell activation and CD8^+^ T cell–mediated antitumor immunity. These interconnected TME features collectively shape ADC sensitivity and resistance and provide a rationale for combination strategies targeting stromal and immune components.

**Figure 4:**
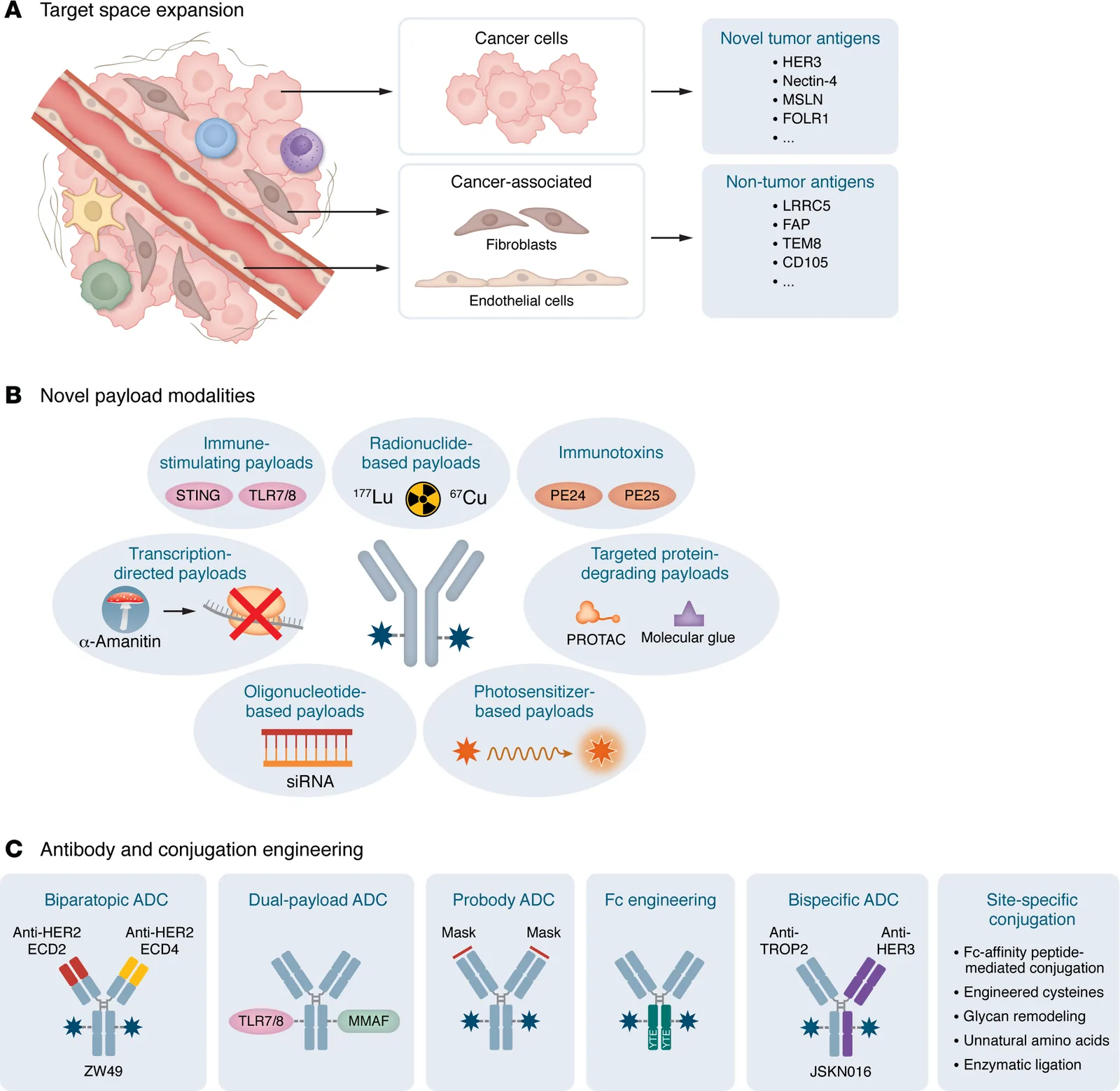
Future generations of ADCs in breast cancer. Next-generation ADC development in breast cancer is advancing along three major axes. (**A**) Target space expansion extends beyond established tumor-associated antigens to include novel tumor cell targets, such as HER3, Nectin-4, mesothelin (MSLN), and folate receptor alpha (FOLR1), as well as nontumor antigens expressed by cancer-associated stromal compartments, including fibroblasts and endothelial cells, exemplified by LRRC5, FAP, TEM8, and CD105. (**B**) Novel payload modalities broaden the functional repertoire of ADCs beyond conventional cytotoxic warheads to include immune-stimulating payloads (for example, STING and TLR7/8 agonists), transcription-directed payloads (for example, α-amanitin), oligonucleotide-based payloads (for example, siRNA), targeted protein-degrading payloads (including proteolysis-targeting chimeras [PROTACs] and molecular glues), immunotoxins (for example, PE24 and PE25), radionuclide-based payloads (for example, ^177^Lu and ^67^Cu), and photosensitizer-based payloads (for example, IR700). (**C**) Antibody and conjugation engineering aims to improve selectivity, stability, and therapeutic index through biparatopic ADCs, dual-payload ADCs, probody ADCs, Fc engineering, bispecific ADCs, and site-specific conjugation strategies, including engineered cysteines, glycan remodeling, Fc-affinity peptide-mediated conjugation, unnatural amino acids, and enzymatic ligation.

**Table 5:**
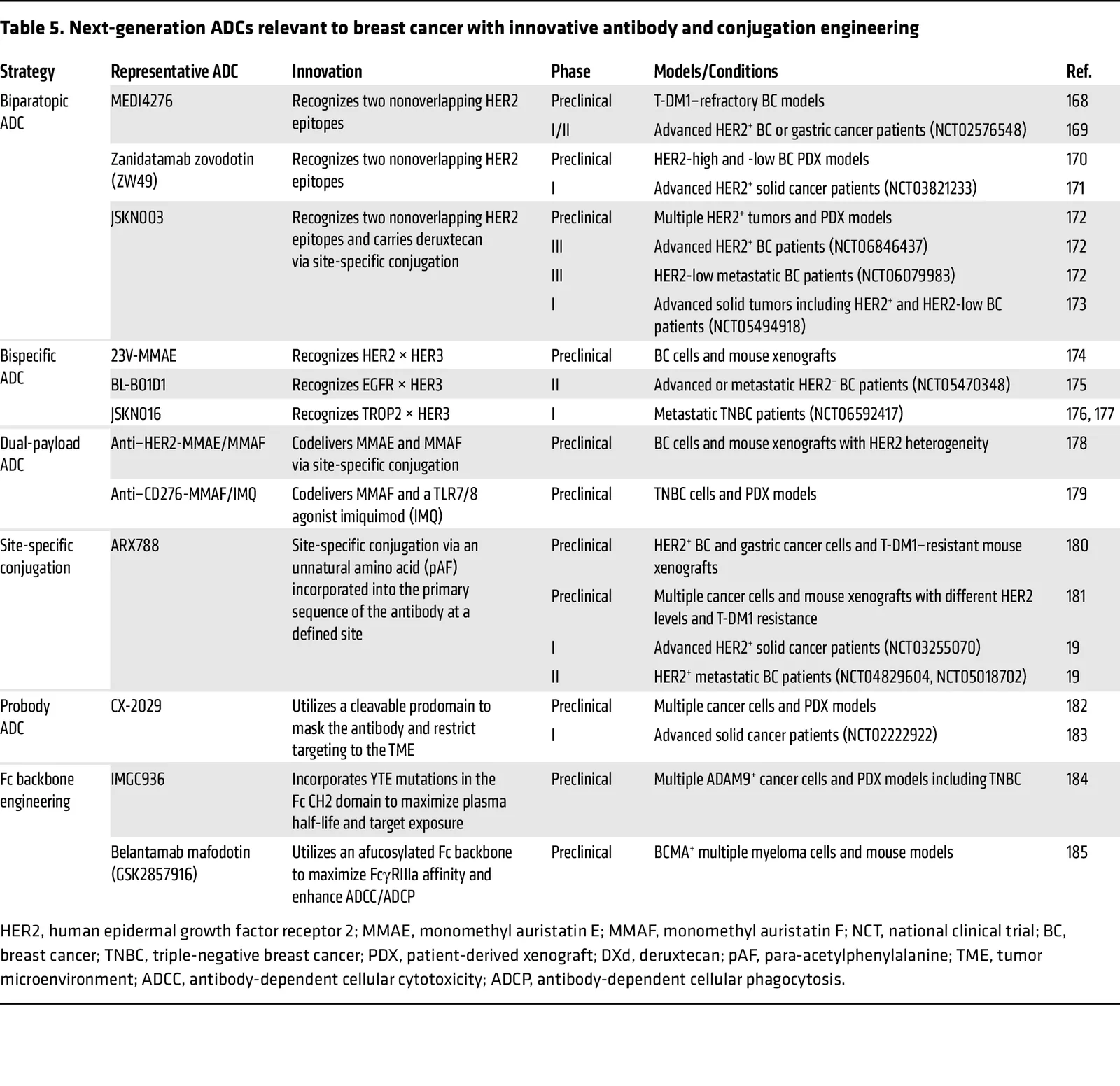
Next-generation ADCs relevant to breast cancer with innovative antibody and conjugation engineering

**Table 4:**
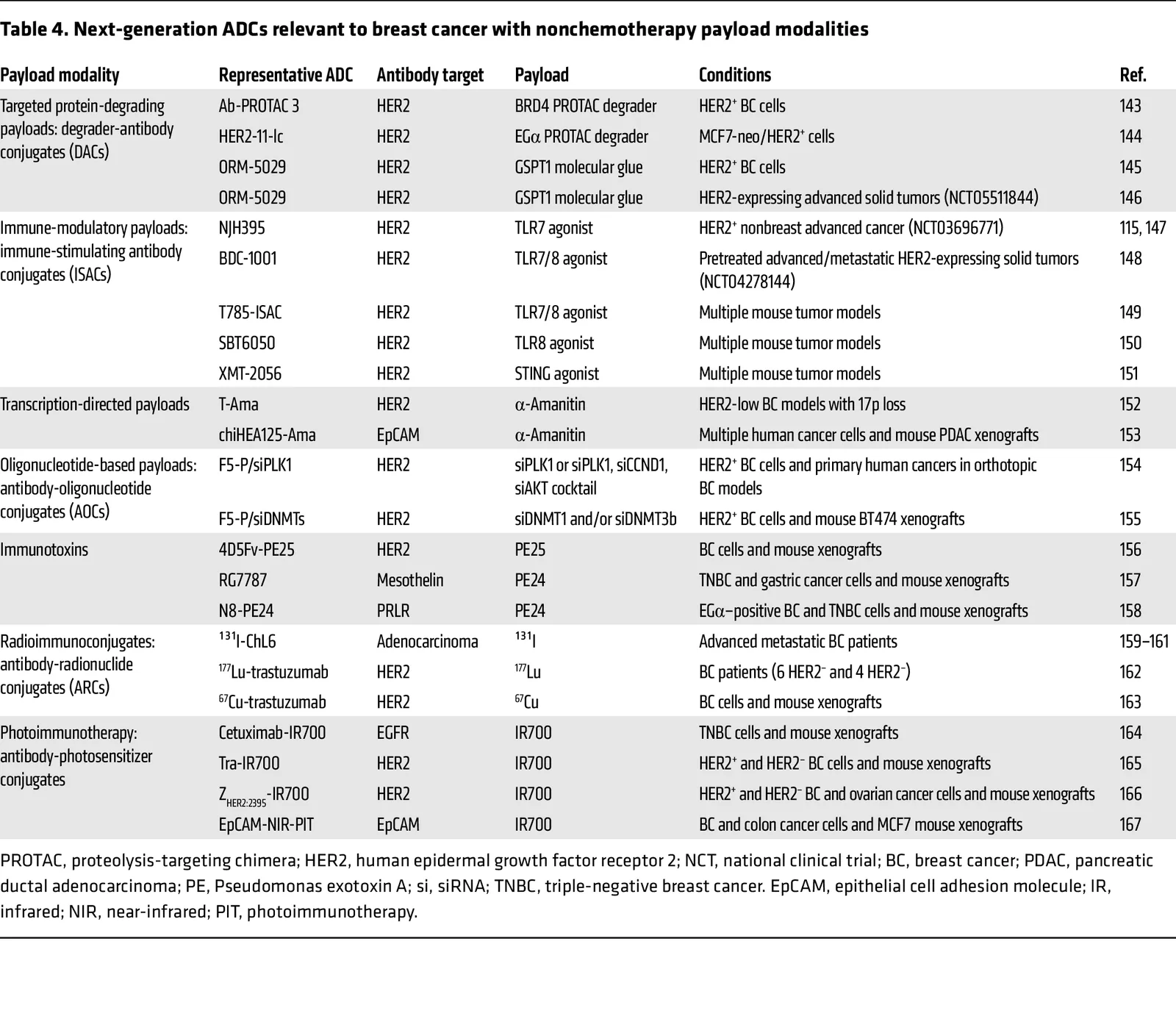
Next-generation ADCs relevant to breast cancer with nonchemotherapy payload modalities

**Table 3:**
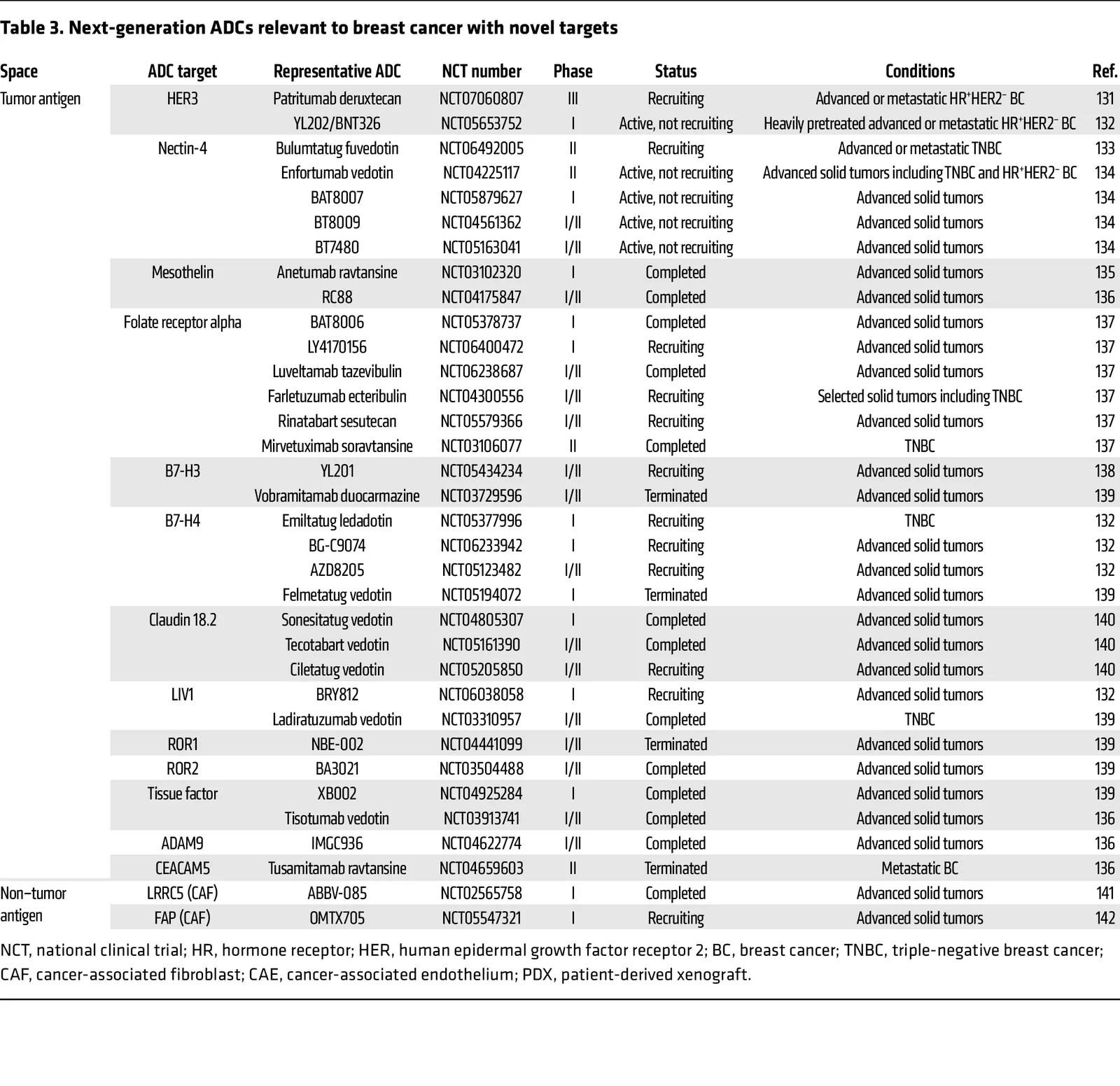
Next-generation ADCs relevant to breast cancer with novel targets

**Table 2:**
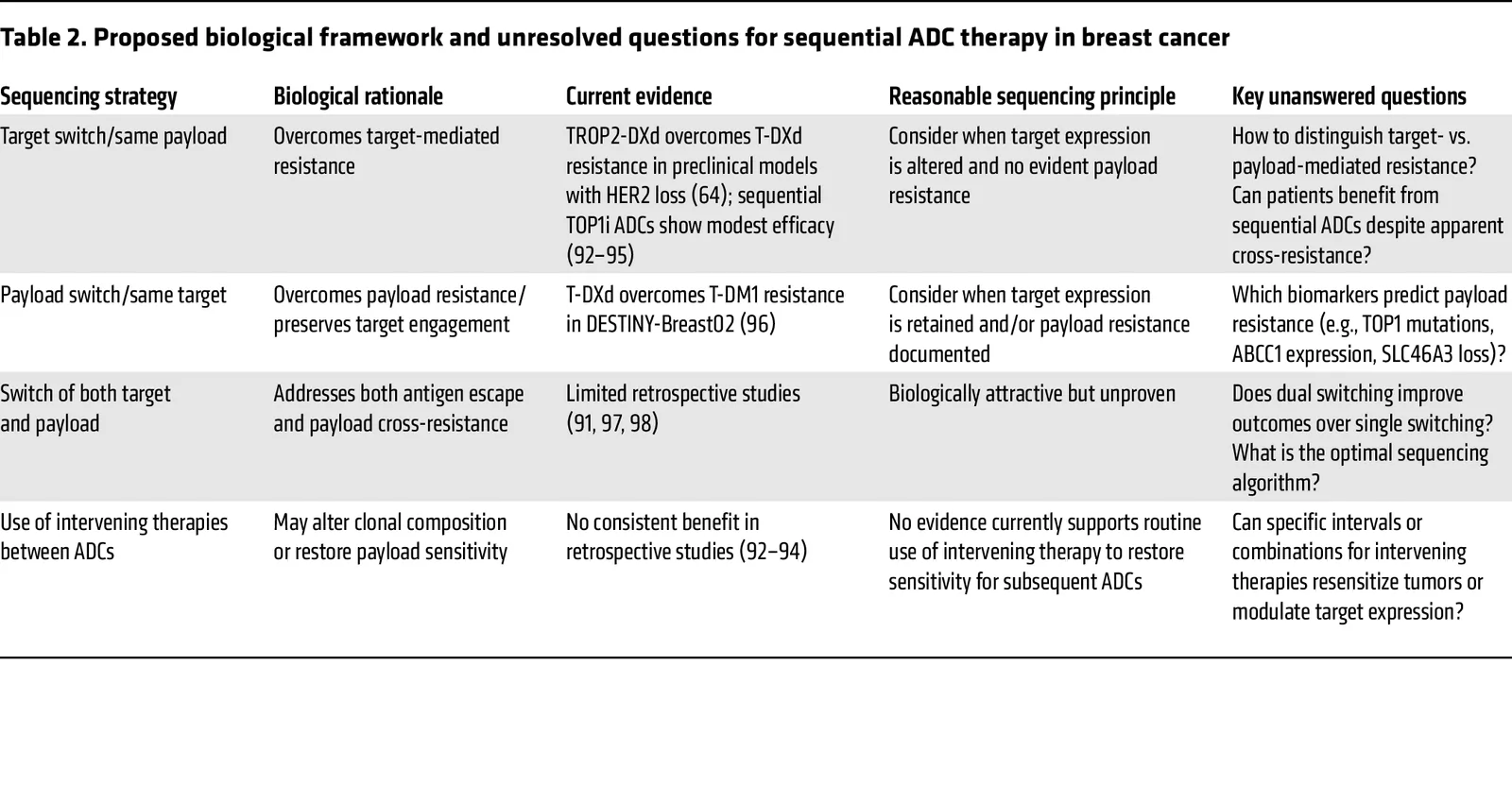
Proposed biological framework and unresolved questions for sequential ADC therapy in breast cancer

**Table 1:**
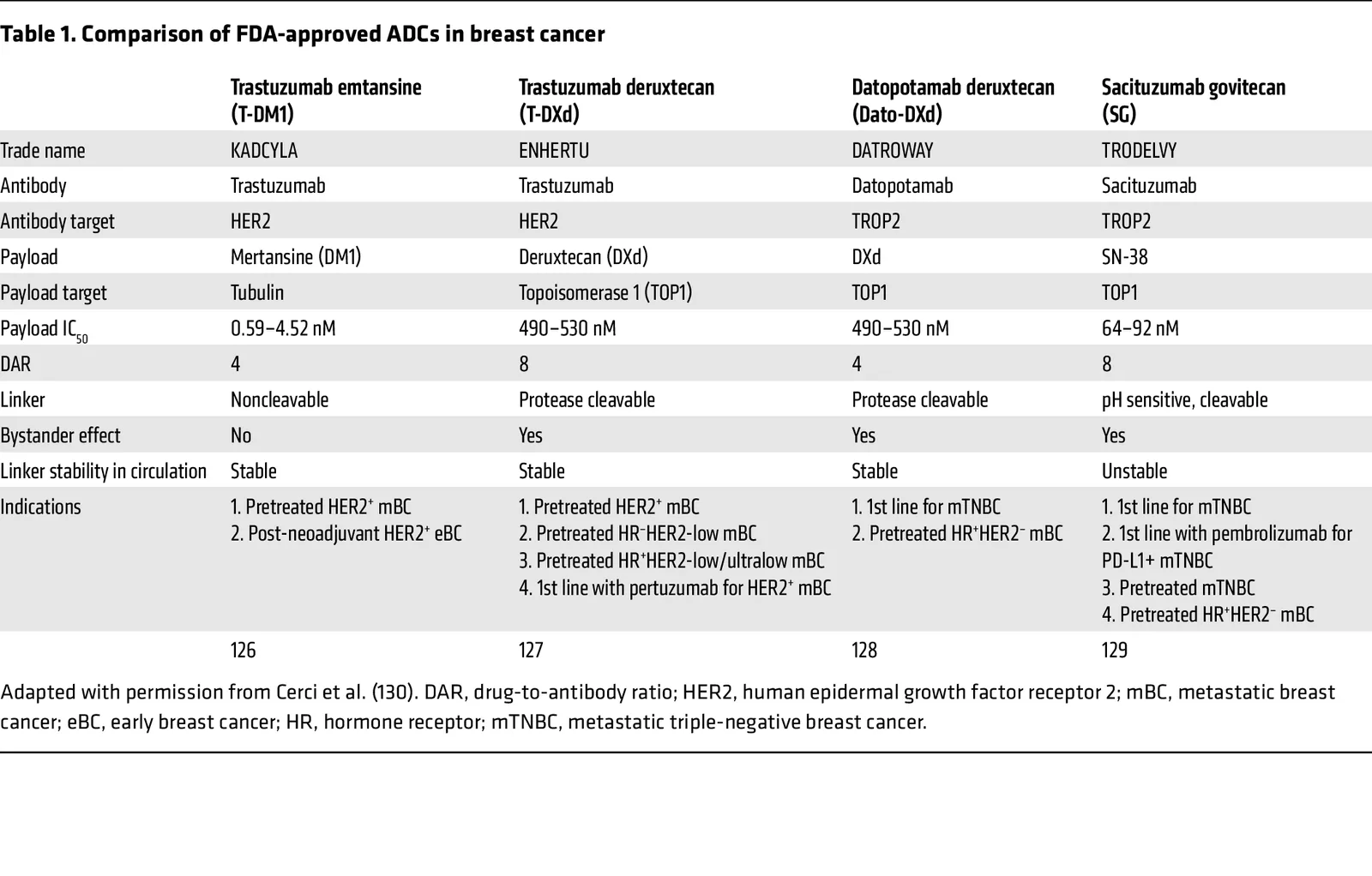
Comparison of FDA-approved ADCs in breast cancer

## Version 1 10/01/2026

Electronic publication
